# “Cave-in” decompression under unilateral biportal endoscopy in a patient with upper thoracic ossification of posterior longitudinal ligament: Case report

**DOI:** 10.3389/fsurg.2022.1030999

**Published:** 2023-01-06

**Authors:** Xiaowei Jing, Zhiyuan Gong, Xiaowen Qiu, Zhuolin Zhong, ZiChuan Ping, Qingfeng Hu

**Affiliations:** Department of Orthopedic Surgery, Fourth Affiliated Hospital of Zhejiang University School of Medicine, Yiwu, China

**Keywords:** thoracic ossification of the posterior longitudinal ligament, cave-in, decompression, unilateral biportal endoscopy, percutaneous endoscopy

## Abstract

**Background:**

Thoracic ossification of the posterior longitudinal ligament (TOPLL) requires surgery for spinal cord decompression. Traditional open surgery is extremely invasive and has various complications. Unilateral biportal endoscopy (UBE) is a newly developed technique for spine surgery, especially in the lumbar region, but rare in the thoracic spine. In this study, we first used a different percutaneous UBE “cave-in” decompression technique for the treatment of beak-type TOPLL.

**Methods:**

A 31-year-old female with distinct zonesthesia and numbness below the T3 dermatome caused by beak-type TOPLL (T2–T3) underwent a two-step UBE decompression procedure. In the first step, the ipsilateral lamina, left facet joint, partial transverse process, and pedicles of T2 and T3 were removed. In the second step, a cave was created by removing the posterior third of the vertebral body (T2–T3). The eggshell-like TOPLL was excised by forceps, and the dural sac was decompressed. All procedures are performed under endoscopic guidance. A drainage tube was inserted, and the incisions were closed after compliance with the decompression scope *via* a C-arm. The patient's preoperative and postoperative radiological and clinical results were evaluated.

**Results:**

Postoperative CT and MR films conformed complete decompression of the spinal cord. The patient's lower extremity muscle strength was greatly improved, and no complications occurred. The mJOA score improved from 5 to 7, with a recovery rate of 33.3%.

**Conclusion:**

UBE spinal decompression for TOPLL showed favorable clinical and radiological results and offers the advantages of minimal soft tissue dissection, shorter hospital stays, and a faster return to daily life activities.

## Introduction

Thoracic ossification of the posterior longitudinal ligament (TOPLL) frequently occurs in Asians, and the incidence of TOPLL is approximately 2.2% in Chinese people (1). Thoracic myelopathy due to TOPLL is rare and is an indication for surgical treatment because of the progressive reduction in lower limb muscle strength.

Several procedures have been performed to treat TOPLL. Anterior decompression is an ideal treatment approach for TOPLL, although it requires a high level of technique to open the thoracic and pleural cavities and has a higher rate of complications (2–4). While posterior decompression is a relatively safe approach, indirect decompression is provided by a backward shift of the spinal cord without removing the TOPLL. The remaining TOPLL could cause insufficient decompression in some clinical situations, such as beak-type TOPLL or severe compression on the ventral spinal cord (5–7). Circumferential decompression *via* a single posterior approach has emerged as a promising surgical procedure because it allows removing the bony compression before the dural sac directly (8–10). However, this approach is quite invasive and has a variety of unexpected postoperative complications because of the prolonged operative duration, more blood loss, and blocked vision during the management of the complex composed of the posterior wall of the vertebral body and the ossified posterior longitudinal ligament (8–10).

UBE is a newly developed technique for lumbar surgery, but is rarely used in thoracic spine disorders. The following report presents a case with beak-type TOPLL that underwent percutaneous UBE “cave-in” decompression. This is the first reported case in the literature of the treatment of TOPLL using the unilateral biportal full-endoscopy technique.

## Materials and methods

### Case presentation

**Figure 1:**
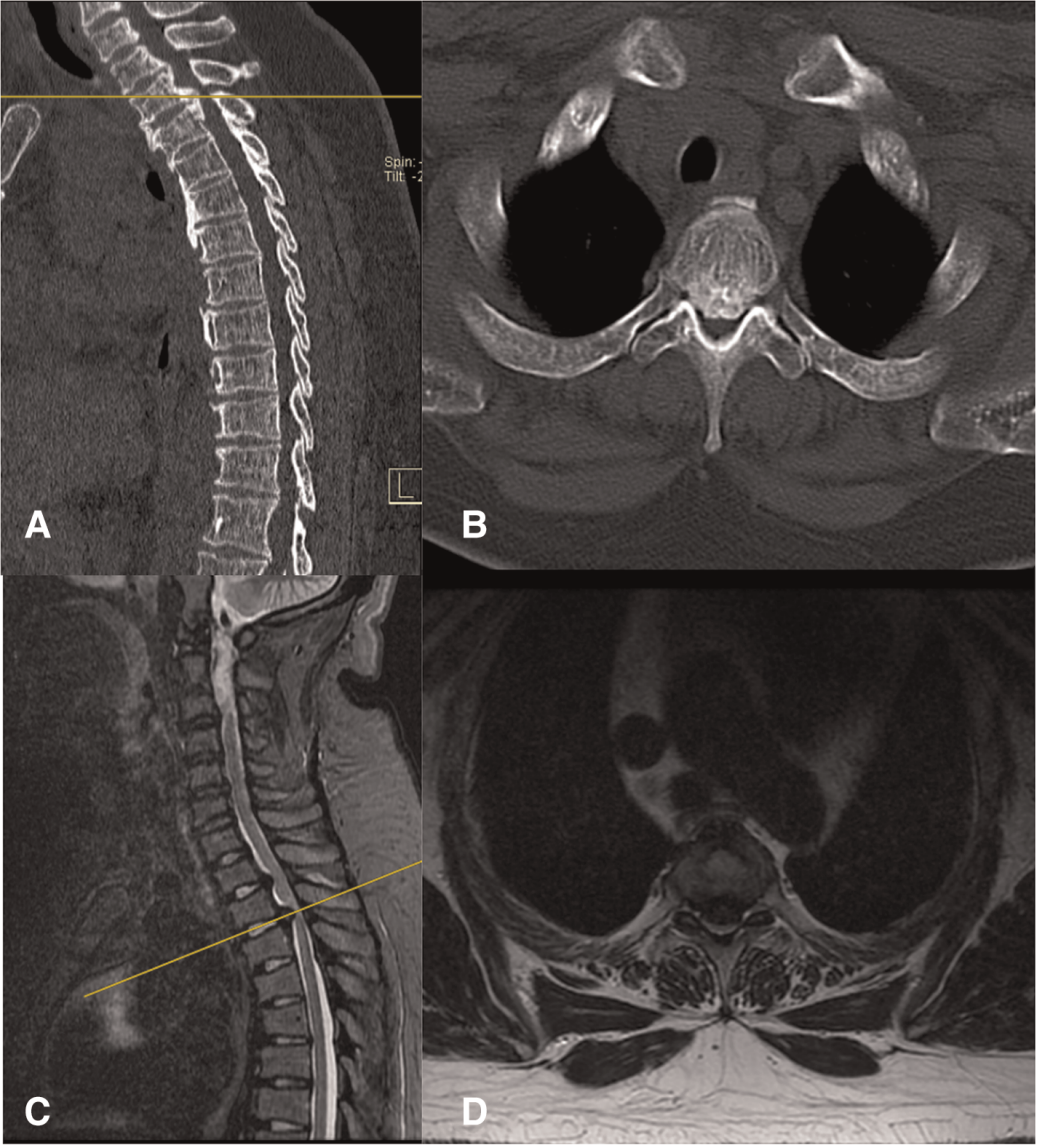
(**A,B**) The preoperative CT films conformed TOPLL at T1–T2 and T2–T3. (**C,D**) The preoperative MRI suggest the spinal cord at T2–T3 was compressed by TOPLL and became thinning.

A 31-year-old female had a 4-year history of gait disturbance and bilateral lower limb weakness, which had aggravated for 11 days. She had pseudopseudohypoparathyroidism and severe constipation and was taking a daily dose of 0.5 mcg of calcitriol and 1,200 mg of calcium carbonate D3 tablets. A neurologic examination identified distinct zonesthesia and numbness below the T3 dermatome. Upper limb muscle strength was expected; however, the lower limb muscle strength was decreased to level 3. Deep tendon reflexes were increased with ankle and knee jerks. Babinski's sign and ankle clonus were positive. According to the Modified Japanese Orthopedic Association (mJOA) Scoring System, the patient scored 5. Sagittal computed tomography (CT) films demonstrated the beak-type TOPLL at T1–T2 and T2–T3 levels, which occupied approximately 65% of the spinal canal volume at T2–T3 (Figures 1A,B). Magnetic resonance imaging (MRI) in the sagittal view showed that the spinal cord was severely compressed on the ventral side of the spinal cord and remarkable degeneration was caused by ossification of the posterior longitudinal ligament (OPLL) at T2–T3 (Figures 1C,D). Based on these findings, thoracic myelopathy (T2–T3) caused by TOPLL was diagnosed.

## Procedure

### Position, incision, instruments, and portals design

**Figure 2:**
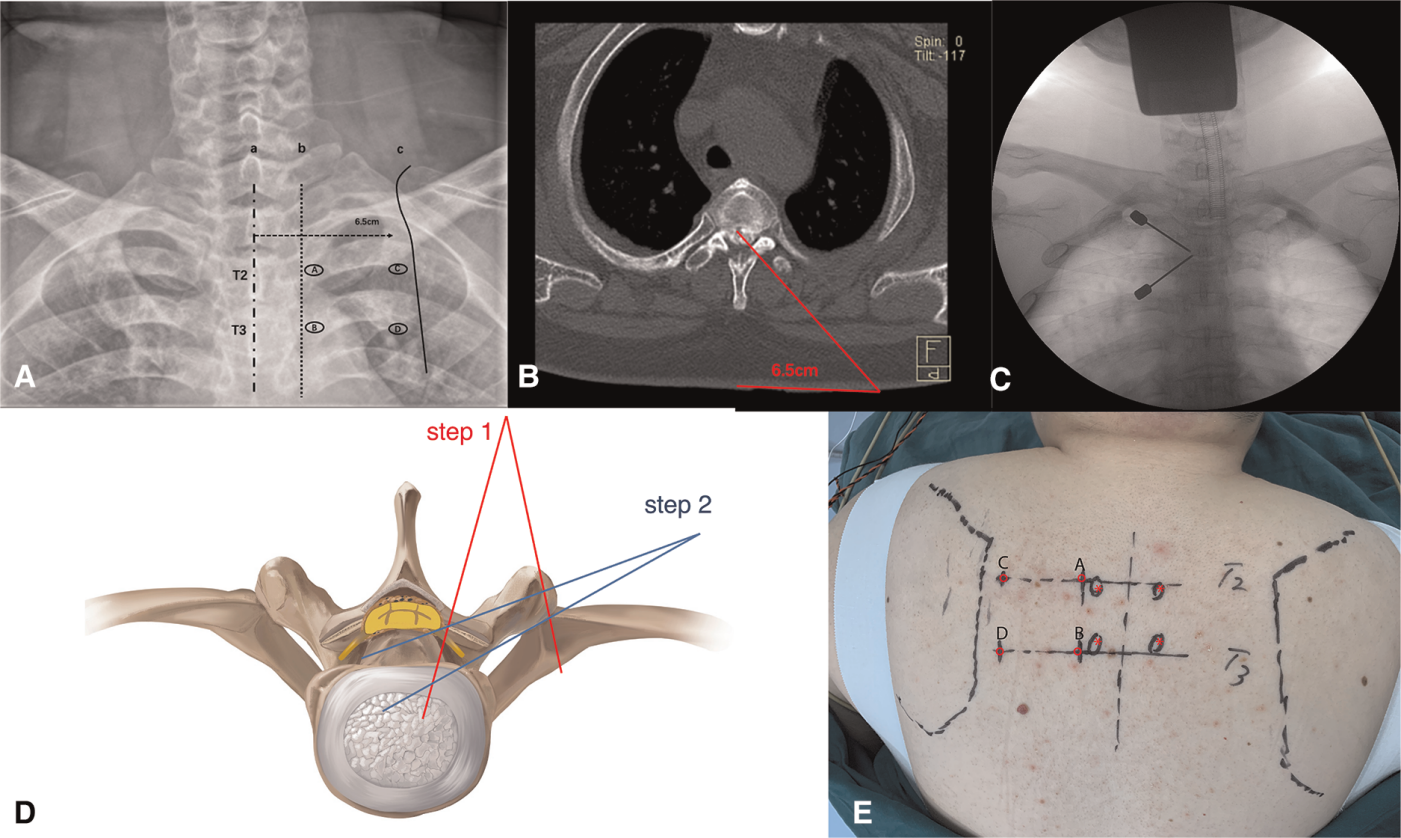
(**A,E**) The localization of the portals on the preoperative anteroposterior radiograph and the body surface. (**B,D**) Preoperative assessment of the extent of spinal cord decompression. (**C**) Puncture at portals A and B, and the target is the left T2–T3 facet joint.

A novel anterior “cave-in” decompression through percutaneous biportal full endoscopy with a posterior approach for local beak-type TOPLL was employed. The decompression process is a two-stage process. All procedures were performed under neurophysiological monitoring. The endoscopy system used in this case was composed of a 30° angled scope with a continuous water irrigation system, power system, and radiofrequency system (BONSY Corporation, Shang Hai, China). The patient was positioned in a neutral prone position on a radiolucent operating table, and the surgery was performed under general anesthesia. Determine the location of the portals and the decompression range of the two steps on the imaging films preoperatively (Figures 2B,D).

The target level and the puncture sites on the skin for the portals were confirmed by C-arm before the operation. Portals A and B were located at the lateral edges of the left pedicles of T2 and T3, respectively. In the same axial plane as portal A, portal C was 6.5 cm away from the posterior median line, which was close to the medial edge of the scapula. The same way to create portal D (Figures 2A,E). Portals A and C were for observation, and portals B and D were for operation.

### Unilateral biportal approach for OPLL excision

**Figure 3:**
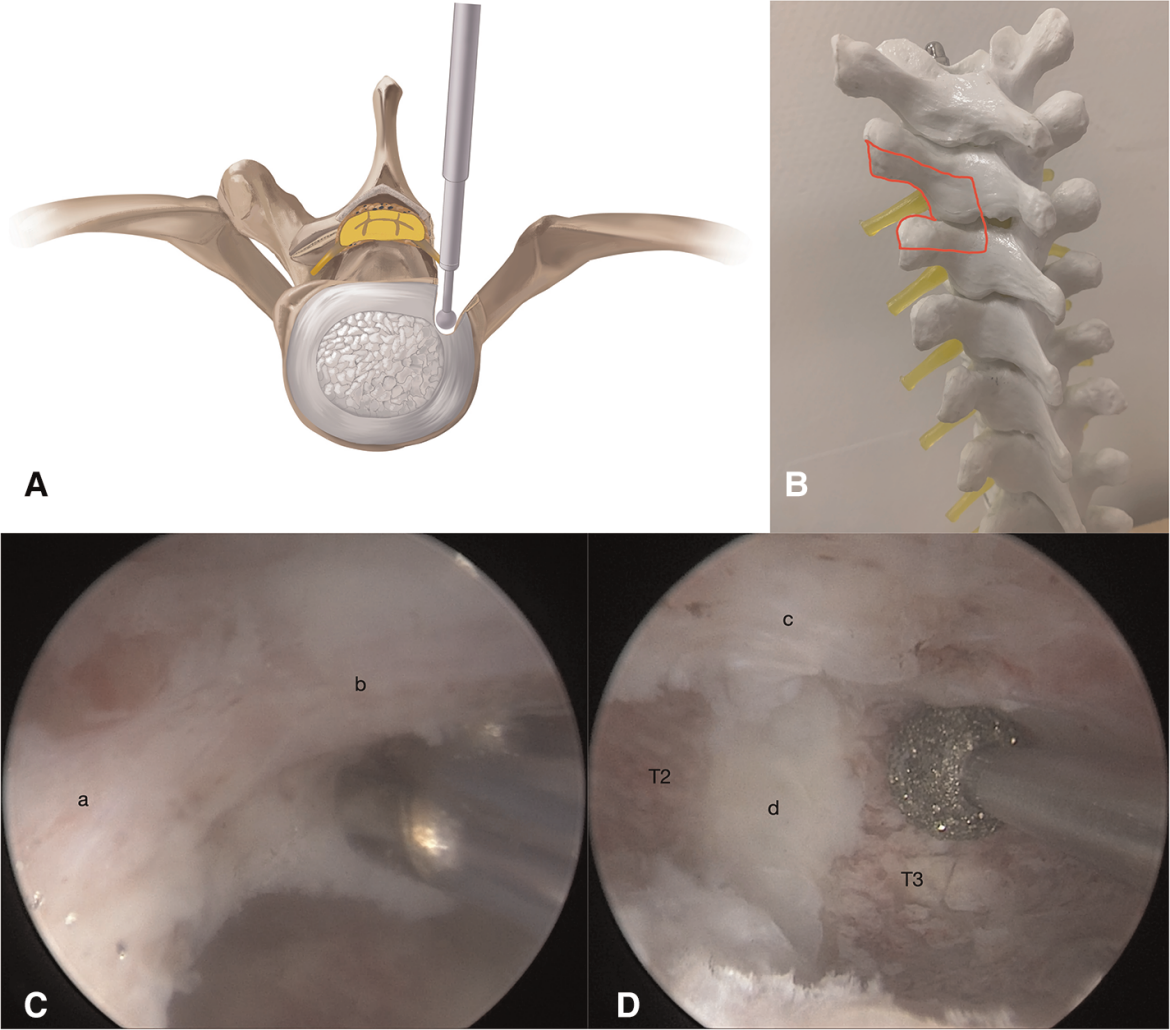
(**A,B**) Illustration showing the drilling of ipsilateral lamina, left facet joint, partial transverse processes, and pedicles of T2 and T3 by step 1. (**C,D**) Exposure of T2 intercostal nerve (a), dural sac (b), TOPLL (c) and intervertebral disc of T2–T3 (d).

The first stage of the procedure was to excise the bony structures, which would have hindered the observation and manipulation during the second stage, and expose the dural sac margin. Puncture at sites A and B with two spinal puncture needles targeting the left facet joint of T2–T3 (Figure 2C). Cut the skin and muscle along with the puncture needles to the bone surface. Sweep the muscle overlying the lamina and facet joint of T2–T3 with a periosteal elevator to create a space sufficient for observation and operation. An arthroscopy irrigation system was inserted through portal A, while conventional spinal surgery instruments and the radio frequency (RF) catheter entered from portal B. The water pressure was under 30 mmHg to avoid excessively increase epidural pressure, which can cause elevated intracranial pressure and spinal cord injury. The RF catheter was used to hemostasis and clean soft tissue to expose the lamina, facet joint, transverse process, and lateral wall of the pedicle (T2 and T3). Then, the ipsilateral lamina, left facet joint, partial transverse processes, and pedicles of T2 and T3 were ground and removed by the arthroscopic 4-mm burr until the margin of the dural sac and intercostal nerve (T2) were exposed (Figure 3).

**Figure 4:**
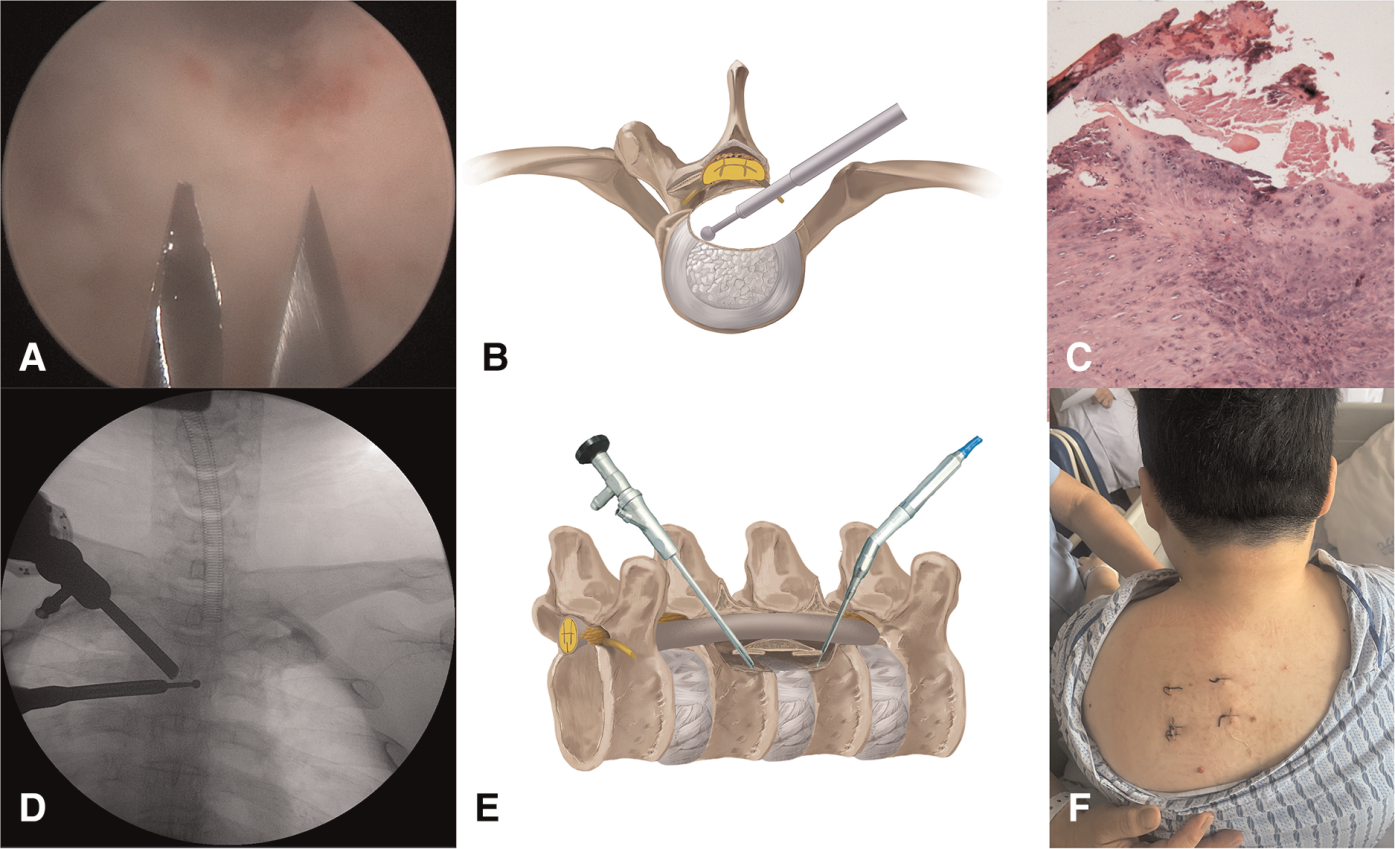
(**A**) Create portals C and D under direct endoscopy view. (**B,E**) Illustration of the “cave” boundaries created by the grinding burr. (**C**) Pathological image of the removed OPLL. (**D**) The grinding burr has reached the medial wall of the pedicle conformed by C-arm. (**E**) Skin incisions of the four portals.

The second stage was to remove the TOPLL and decompress the spinal cord. Create portals C and D with two K wires under endoscopic supervision (Figure 4A). The TOPLL and posterior portions of the vertebral bodies were partially resected using the high-speed drill (Supplementary Video 1). A cave was created from left to right until the TOPLL was separated from the vertebral body, then the left eggshell-like TOPLL sticking to the ventral dural sac was released (Figures 4B,E). The L-shape hook and nerve stripper were used to separate the TOPLL and dural sac, followed by the removal of the TOPLL piece by piece using forceps (Supplementary Video 2). The separation process must be performed gently and carefully to avoid cerebrospinal fluid leakage due to the rupture of the dural sac or even iatrogenic spinal cord injury. Postoperative pathology confirmed that the excised osteophyte was composed of cartilage and bone tissue (Figure 4C). Endoscopic visualization of the grinding burr touching the contralateral cavity wall shows that a C-arm machine was used to determine the extent of decompression (Figure 4D). A drainage tube was placed through portal D, and all four portals were closed by sutures (Figure 4F).

## Results

**Figure 5:**
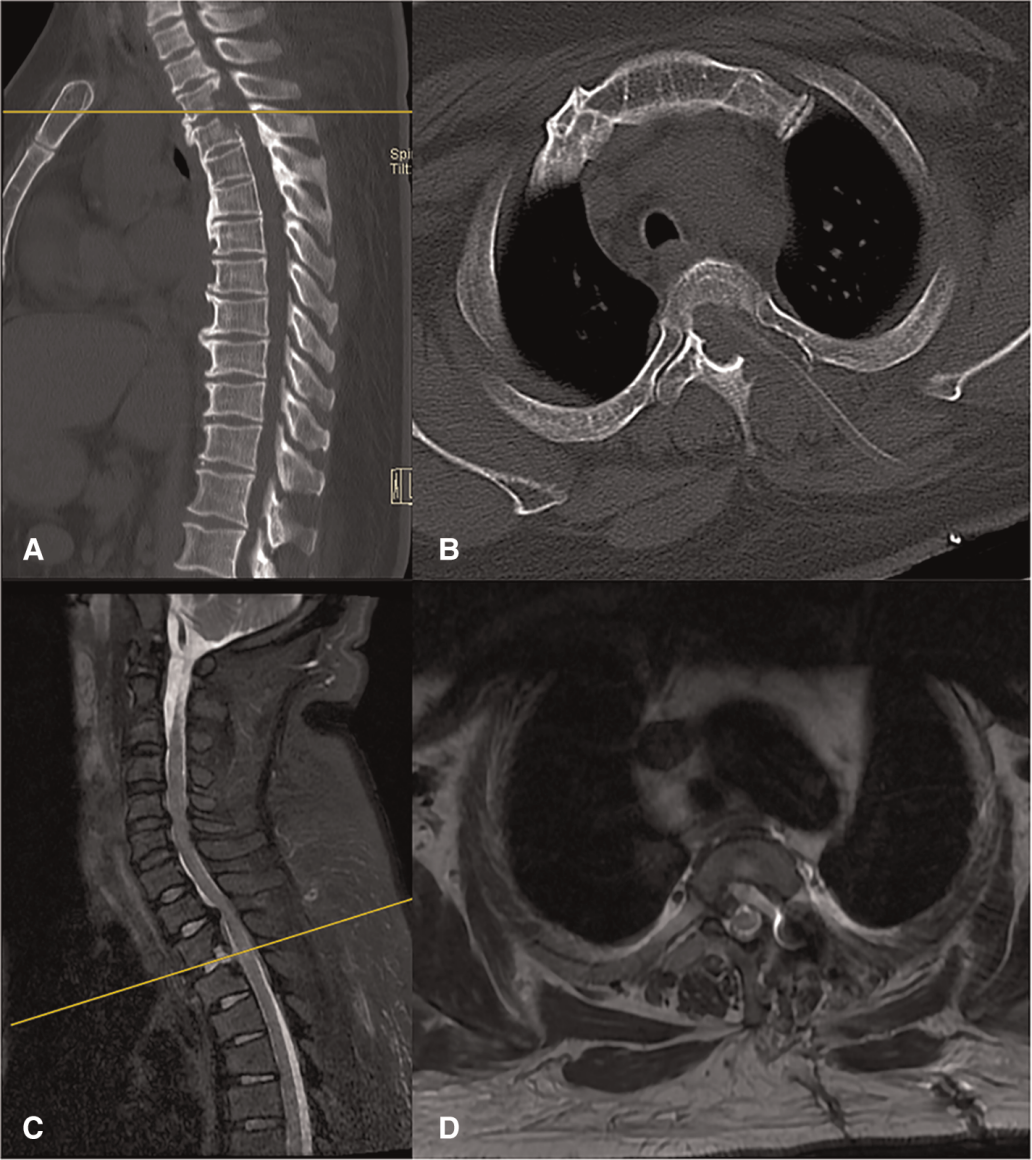
Postoperative CT (**A,B**) and MRI (**C,D**) showed the most TOPLL was excised and the thoracic spinal cord was completely decompressed.

**Figure 6:**
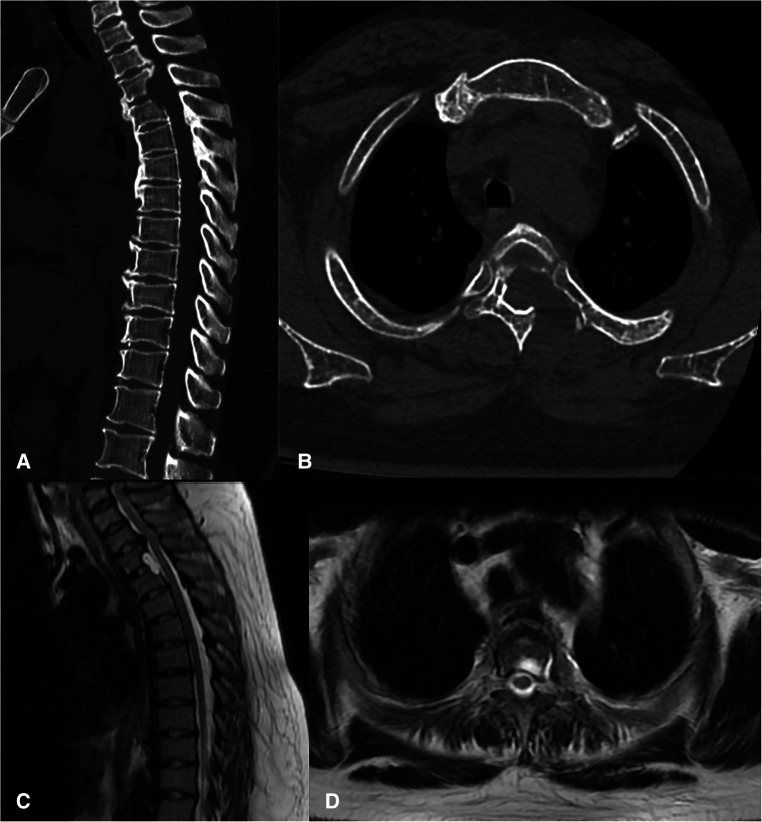
There was no evidence of instability or kyphosis on CT (**A,B**) and MRI (**C,D**) films 6 months after surgery and the thoracic spinal cord was completely decompressed.

The duration of the surgery was 3.5 h, and there were no postoperative complications such as cerebrospinal fluid leakage and decreased muscle strength. The patient made a quick recovery and started postoperative ambulation after the drainage tube was removed 36 h after surgery. Lower limb function was significantly improved. Moreover, constipation has been significantly relieved. The postoperative modified JOA score was 7, and the recovery rate was 33.3%. Postoperative CT and MR images showed that the OPLL was removed, and the spinal cord was completely decompressed (Figure 5). There was no evidence of instability or kyphosis on CT and MRI films 6 months after surgery (Figure 6).

## Discussion

TOPLL might cause thoracic spinal stenosis and the compression of the ventral part of the spinal cord. The patient exhibits progressive deterioration of motor and sensor function in the lower limbs, accompanied by excretory dysfunction (12). Anterior direct decompression by excision of OPLL bone blocks before the spinal cord is an ideal surgical approach for the patient with OPLL occupying more than 50% of the canal, especially the beak-type OPLL. The approach to the upper thoracic vertebral bodies involves the trachea, esophagus, and vital vascular and neural structures and requires complicated operation processes (13). One-stage posterior circular decompression of the thoracic spine has been widely recognized because of its effectiveness in removing the compression around the spinal cord in patients with OPLL and/or ossification of the ligamentum flavum (14). However, this approach is extremely invasive and can be result in postoperative complications such as intercostal nerve palsy, neurological deterioration, and cerebrospinal fluid leakage (15).

To the best of our knowledge, the use of UBE technology for TOPLL through a posterolateral approach has not been reported. The unilateral biportal endoscopy (UBE) technique has been used in degenerative diseases of the lumbar spine for the past few years (16–19). The total number of articles published about UBE has increased in a straight line since 2007, which indicates that spine surgeons are paying more attention to the field (20). Open circumferential decompression for TOPLL through a posterior approach carries the risk of complications because of the blinded resection of the lesion and its positioning ventrally to the dura mater (8). In our patient, we decompressed the spinal cord by removing the ventral OPLL under direct visualization guided by the “cave-in” decompression theory. Under the sight of the 30° angled endoscopy, the dissection of TOPLL from the ventral dura was unblinded and safer. Likewise, Baram excised the TOPLL under the visualization conditions through the traditional posterior one-stage 360° circumferential decompression with the assistance of a 60° angled endoscopy (21). UBE for the treatment of TOPLL carries risks; therefore, the surgeon needs to develop expertise and additional experience in arthroscopic operations or UBE lumbar procedures. Portal design and decompression range should be well planned on the radiology films.

Over the past few years, spine surgeons have applied endoscopic surgical techniques mastered in the lumbar spine to the treatment of thoracic pathology. Gibson reviewed the literatures on the full endoscopy treatment of thoracic stenosis and disc prolapse from 2000 to 2020, and the majority were treated by the transforaminal approach and the interlaminar approach (22). However, most of them are reported on disc pathology or ossification of ligamentum flavum, with few studies about TOPLL (23, 24). Yu et al. presented a series of cases with single-level TOPLL that underwent full endoscopy uniportal decompression *via* a transforaminal approach and believed that the thoracic endoscopic technique is an effective and safe alternative approach for conventional procedures (25). Kong performed uniportal endoscopy decompression for TOPLL at the T1–T2 level through a transcorporeal approach, and postoperative MRI and CT showed that the major part of the OPLL was removed and the spinal cord compression was relieved (26). However, the current literature remains limited to level IV evidence, despite the fact that the patients with TOPLL treated by endoscopy decompression in the previous studies have achieved favorable clinical outcomes. In the future, retrospective studies with more patients or even randomized controlled studies will be needed to provide higher level evidence.

The patient in this study did not experience instrumentation. The vertebral body, intervertebral disc, facet joints, and costovertebral complex are considered important structures for the stabilization of the thoracic spine. Patients with TOPLL treated with multilevel posterior decompression have developed neurological deterioration caused by kyphosis and instability (27, 28). Unlike the previously reported methods, we just removed the unilateral facet joint, and a small portion of the vertebral body, the intervertebral disc, the unilateral pedicle, and the transverse process. The posterior ligamentous complex was completely preserved in this technique. Additionally, T2 and T3 were bridged by the osteophyte generated before the T2–T3 disc, which means the two vertebral bodies had fused spontaneously (Figure 1). Although there is less risk of instability and kyphosis, we still inform the patient return visit on time. Yoon reports a case with TOPLL treated through a lateral transthoracic approach by drilling the whole rib head, part of the pedicle and upper vertebral lamina, the posterior one-third intervertebral disc, and the vertebral body (29). The excision range of bony structures is similar to ours, and there was no kyphotic change or instability in the thoracic spine after more than a year of follow-up, although instrumentation was not performed. Biomechanical studies suggested thoracic spine stability was not signiﬁcantly affected by sequential decompressive procedures consisting laminectomy, unilateral facetectomy, and unilateral costotransversectomy in thoracic segments at the level of the true ribs in all three planes of motion (30, 31).

There are some tips that surgeons should not overlook. The distance to the posterior media line of portals C and D should be identified on the CT film before surgery in order to remove most of TOPLL without stimulating the dural sac. Puncture of portals C and D with two K wires should be done under endoscopic supervision, and the procedure requires a high level of hand-eye coordination to avoid pleural injury. We suggest starting by grinding from the lateral edge of the facet joint. Then, exposure of the lateral edge of the dural sac could help us identify the “safety zone” for the next grinding work (Supplementary Video 3). The cave was finished when the left eggshell OPLL was completely freed from the vertebral body, followed by stripping the OPLL from the ventral dura piece by piece. As for intraoperative bleeding control, we recommend the use of radiofrequency for hemostasis of soft tissue. During the operation, maintain blood pressure between 100 and 110 mmHg and the use of an emery grinding head could control the seeping of blood from the surface of the bone. Bone wax was used to seal the surface of the vertebral cancellous bone and stop the intractable punctate hemorrhage.

In summary, the removal of TOPLL by percutaneous UBE successfully alleviated the symptoms of the patients. This approach has significant advantages over the traditional open approach because of the preservation of the posterior ligamentous complex. In particular, its lower risk, smaller incision, shorter hospital stay, and faster return to daily life activities make it an attractive surgical option.

## Data Availability

The original contributions presented in the study are included in the article/**Supplementary Material**, further inquiries can be directed to the corresponding author/s.
